# Announcement

**Published:** 2015-01-16

## Updates to the Overseas Immunization Program for United States–Bound Refugees

Refugees being resettled in the United States, unlike immigrants seeking residency, have not been subject to immunization requirements (1). Without immunization, refugee communities overseas and in the United States are vulnerable to outbreaks of vaccine-preventable diseases that can disrupt the resettlement process and require costly public health responses (2,3). CDC’s Division of Global Migration and Quarantine has regulatory authority to prevent communicable disease importation among the approximately 70,000 refugees resettled in the United States each year.

Historically, logistical challenges prevented overseas routine vaccination of refugees scheduled for resettlement in the United States. However, in December 2012, CDC began implementation of an overseas program that resulted in the routine vaccination of United States–bound refugees in six countries: Thailand and Nepal (initiated December 2012), Malaysia and Kenya (initiated September 2013), Ethiopia (initiated November 2013), and Uganda (initiated August 2014). Refugees vaccinated through this program began arriving in the United States in 2013. The program covers approximately 50% of refugees who arrive in the United States annually and likely will be expanded to include countries from which other refugees originate.

A collaboration with two other agencies (the U.S. State Department’s Bureau of Population, Refugees, and Migration and the International Organization for Migration), the overseas vaccination program is intended to reduce U.S. disease outbreaks by ensuring that refugees arrive in the United States protected against vaccine-preventable diseases. Depending on age and individual risk factors, refugees now receive 2 to 3 doses of the following vaccines while overseas: polio; measles, mumps, and rubella; hepatitis B; pneumococcal conjugate; and *Haemophilus influenzae* type B. Initial doses are given during the immigration medical examination 2–6 months before departure for the United States. These vaccines were selected after considering disease risk and the cost and availability of the vaccines in refugee camp settings.

Information on participating countries and current vaccine schedules is available at http://www.cdc.gov/immigrantrefugeehealth/guidelines/overseas/interventions/immunizations-schedules.html, or by contacting CDC at dgmqpdi@cdc.gov. Vaccines administered to refugees through this program are documented in the Division of Global Migration and Quarantine’s Electronic Disease Notification System and are accessible to clinics conducting postarrival refugee medical examinations (4). More information on the notification system is available by contacting the help desk at edn@cdc.gov.
